# Efficacy of Sirolimus Treatment in PEComa–10 Years of Practice Perspective

**DOI:** 10.3390/jcm10163705

**Published:** 2021-08-20

**Authors:** Tomasz Świtaj, Aleksandra Sobiborowicz, Paweł Teterycz, Anna Klimczak, Donata Makuła, Michał Wągrodzki, Anna Szumera-Ciećkiewicz, Piotr Rutkowski, Anna M. Czarnecka

**Affiliations:** 1 Department of Soft Tissue/Bone, Sarcoma and Melanoma, Maria Sklodowska-Curie National Research Institute of Oncology, 02-781 Warsaw, Poland; tswitaj@coi.pl (T.Ś.); a.sobiborowicz@cent.uw.edu.pl (A.S.); pawel.teterycz@pib-nio.pl (P.T.); Anna.Klimczak@pib-nio.pl (A.K.); piotr.rutkowski@pib-nio.pl (P.R.); 2 Medical Faculty, Medical University of Warsaw, 02-091 Warsaw, Poland; 3 Department of Computational Oncology, Maria Sklodowska-Curie National Research Institute of Oncology, 02-781 Warsaw, Poland; 4 Department of Radiology I, Maria Sklodowska-Curie National Research Institute of Oncology, 02-781 Warsaw, Poland; Donata.Makula@pib-nio.pl; 5 Department of Pathology and Laboratory Diagnostics, Maria Skłodowska-Curie Institute-Oncology Center, 02-781 Warsaw, Poland; michal.wagrodzki@pathologist.cc (M.W.); Anna.Szumera-Cieckiewicz@pib-nio.pl (A.S.-C.); 6 Department of Diagnostic Hematology, Institute of Hematology and Transfusion Medicine, 00-791 Warsaw, Poland; 7 Department of Experimental Pharmacology, Mossakowski Medical Research Centre, Polish Academy of Sciences Warsaw, 02-106 Warsaw, Poland

**Keywords:** perivascular epithelioid cell tumor, PEComa, lymphangioleiomyomatosis, sarcoma, sirolimus, mTOR inhibitors

## Abstract

Perivascular epithelioid cell tumors (PEComa) represent a family of rare mesenchymal tumors resultant from deregulation in mTOR pathway activity. The aim of this study is to evaluate the long-term efficacy of targeted PEComa treatment. We reviewed all consecutive patients with PEComa who started systemic treatment with sirolimus in our reference sarcoma center between January 2011 and August 2020. Histopathology of PEComa was reviewed and confirmed in all cases by a designated sarcoma pathologist. Any surviving progression-free patients were censored at the last follow-up (31 March 2021). Survival curves were calculated according to Kaplan–Meier method and compared with the log-rank test or a Cox proportional hazard model. Fifteen (12 females and 3 males) consecutive PEComa patients were treated. The median age of patients treated systemically was 50 years. Median progression-free survival (PFS) was 4.9 months (95% CI: 3.8-NA) for first-line chemotherapy and was not reached (95% CI: 42.0-NA) for sirolimus as first-line therapy. There was one objective response (OR) in the chemotherapy group. The OR rate reached 73% (11/15 cases) for sirolimus regardless of the treatment line. All patients archived disease control. Three patients died due to disease progression after 55, 32, and 32 months since metastatic disease diagnosis. After a median follow-up of 55.7 (range: 3.2–220) months, the 5 yr OS was 65% (CI 95% 39–100). Our study is the largest single-institution report on PEComa systemic targeted therapy and fills the gap in the field of advanced PEComa care since the FDA/EMEA approval of sirolimus.

## 1. Introduction

Perivascular epithelioid cell tumors (PEComa) are rare mesenchymal tumors composed of epithelioid cells characterized by histological and immunohistochemical evidence of both smooth muscle and melanocytic differentiation. The PEComa family includes angiomyolipomas (AML), lymphangioleiomyomatosis (LAM), clear-cell sugar tumors (CCST)—pulmonary and extrapulmonary (PEST, primary extrapulmonary sugar tumour), clear-cell myomelanocytic tumors (CCMMT), and primary cutaneous PEComas (CCCMT, cutaneous clear cell myomelanocytic tumors), as well as PEComa NOS (not otherwise specified) tumors [1]. PEComa NOS is a joint term for a broad group of tumors with perivascular epithelioid differentiation, not qualifying for the specific subtype. PEComa family tumors are rare (up to 1 case per 4 million population) and usually occur sporadically. Radical resection may be the curative treatment of most PEComa cases [2,3]. Nevertheless, selected cases show malignant behavior with infiltrative growth, local recurrences after surgical resection, and/or metastatic spread [2,4]. These include renal and extrarenal epithelioid AMLs, extrapulmonary LAM, and malignant PEComa NOS and require a multidisciplinary therapeutic approach, including systemic treatment [5]. No effective chemotherapy for malignant PEComa has been described. Over recent years, significant progress in the understanding of molecular events underlying PEComa development has been achieved. Therefore, the first effective systemic treatment of PEComa tumors was developed based on their underlying biology with deregulation in mTOR pathway activity [4,6]. 

PEComas, especially bilateral renal AMLs and LAM, are among typical physical manifestations of tuberous sclerosis complex (TSC) called Bourneville–Pringle disease. TSC is an autosomal dominant genetic syndrome caused by inactivating mutations of *TSC1* (hamartin gene) and *TSC2* (tuberin gene), characterized by the development of PEComas along with hamartomas, giant cell astrocytomas, and neurologic dysfunction including epilepsy or intellectual disability [7]. Sporadic PEComas were also found to carry *TSC1* or *TSC2* somatic inactivating mutations [8,9,10]. Moreover, in 10–20% of sporadic PEComas cases, loss of 9q34 (*TSC1*) or 16q13.3 (*TSC2*) has been reported [11]. Tuberin and hamartinregulate mTOR pathway, and hamartin forming a complex with tuberin, stabilize it and protecting against proteosomal degradation. Their loss results in high mTOR activity with increased ribosomal biogenesis, translation, pentose phosphate pathway, lipid synthesis, glycolysis, cell growth, and proliferation, angiogenesis. [12]. The hyperactivation of mTOR signaling enables PEComa cells to sustain proliferation even in the limited supply of nutrients and growth factors [13] that leads to a lack of cell proliferation inhibition, increased cell migration, and differentiation of PEComa cells [14]. It was shown that inactivating mutations in *TSC1* or *TSC2* (or activating mutations in mTOR) correlate with sensitivity to rapalogs, including sirolimus [15,16]. 

Molecular pathology data has provided a rationale for mTOR inhibitor use in patients suffering from PEComa tumors with and without concomitant tuberous sclerosis [17,18,19]. The first report on inhibition of mTOR complex resulting in modest and transient improvement in lung function and reduction in the size of AML covered 25 patients with LAM and AML treated with sirolimus [20]. Subsequent case reports on the efficacy of rapamycine and sirolimus in patients with tuberous sclerosis complex related PEComas (AML and LAM) were published [21,22,23]. Later the efficacy of sirolimus in 46 LAM cases was established by a randomized, double-blind, phase 3 multicenter international lymphangioleiomyomatosis efficacy and safety of sirolimus (MILES) trial [24]. Concordantly several clinical trials of the use of mTOR inhibitor–everolimus in patients with tuberous sclerosis were performed, yielding positive results and leading to registration of everolimus to treat high-risk renal AMLs [25,26]. In the largest, randomized, double-blind, phase 3 EXIST-2 trial, treatment response (defined as a decrease in AML mass by at least 50%) was observed in 42% of patients receiving everolimus [27]. Due to the low incidence of PEComa in the general population, reports on the use of sirolimus in this population of patients are limited. Most case series that have been published report on the efficacy of surgery in PEComa patients [3,28,29]. The largest case series from Royal Marsden Hospital covered ten consecutive patients treated with sirolimus or temsirolimus between 2007 and 2013 [30]. We aimed to analyze the long-term efficacy of sirolimus usage in routine clinical practice in a national reference sarcoma center. The secondary aim of our analysis was to describe clinical factors correlating with treatment duration and patients’ survival. 

## 2. Materials and Methods

### 2.1. Analyzed Group

We included in the analysis consecutive patients affected by advanced, metastatic PEComa treated in the Maria Sklodowska-Curie National Research Institute of Oncology (MSCNRIO, Warsaw, Poland), the only multidisciplinary sarcoma treatment center in Poland, and therefore the Polish national reference sarcoma center. Patients included in the analysis started treatment between 1 January 2011 and 31 August 2020. The follow-up data cut-off was 31 March 2021. Specific inclusion criteria were: (1) male or female patients ≥18 years of age, (2) histologically confirmed diagnosis of PEComa, (3) available formalin-fixed paraffin-embedded (FFPE) sample from core needle biopsy, (4) available CT scan at treatment start, (5) and mTOR inhibitor systemic treatment history. There was no significant family history of other cancers in any of the patients. Tuberous sclerosis complex was excluded based on clinical diagnostic criteria [31].

The histopathology diagnosis of all enrolled patients was reviewed in MSCNRIO by experienced sarcoma pathologists as reported by us before [32], including staining with SMA, Desmin, h-caldesmon, S100p, SOX-10, CD34, ERG, CKAE1/AE3. Cathespin K, HMB-45. Melan A, MITF, TFE3, CD163, Ki-67. Tumor slices from all patients demonstrated strong, diffuse, cytoplasmic staining for phosphorylated S6 protein as per activation of mTORC1. 

### 2.2. Treatment

We have analyzed patients who were ineligible for surgery and were treated systemically in accordance with national sarcoma treatment guidelines [33,34,35]. Treatment breaks and dose reductions were implemented for moderate or severe toxicity by attending physician choice. Therapeutic drug monitoring was performed each cycle. A whole-blood sirolimus therapeutic window of 5 to 15 ng/mL as measured by HPLC/immunoassay was used for safety evaluation [36]. For blood levels above 20 ng/mL dosing was reduced, as also described by others [37]. 

Disease stage and progression were assessed by contrast-enhanced CT scans at baseline and at 3-month intervals or as recommended by the attending physician. Patients were treated continuously until disease progression (PD) or unacceptable toxicities. RECIST v.1.1 criteria were used to assess the effects of sirolimus in this cohort [38].

### 2.3. Analyzed Data

Patients’ electronic medical records in CGM CLININET HIS (CompuGroup Medical Poland Ltd., Lublin, Poland) were screened with MedStream Designer (MSD) software (Transition Technologies, Łódź, Poland). The corresponding 10th revision of the International Statistical Classification of Diseases and Related Health Problems (ICD) C48-C49 and the keyword “PEComa” or “Perivascular epithelioid cell neoplasm” were used. Data were reviewed independently by two researchers. Data of death was confirmed in the Polish National Cancer Registry at the Department of Epidemiology and Cancer Prevention (http://onkologia.org.pl/) via the personal identification number of the patients at 31 March 2021.

### 2.4. Statistical Analysis

The continuous variables were summarized by the median with interquartile range and mean with standard deviation. Categorical variables were described by the count and frequency distribution. All point estimates were reported with a 95% confidence interval (CI). The Kaplan–Meier estimator, the log-rank test, and Cox proportional hazard model were used for the survival and prognostic factors analysis. The median follow-up time was calculated using the reverse Kaplan–Meier estimator. All analyses were performed in the R language version 3.6.3 (The R Foundation for Statistical Computing) with the use of tidyverse and survminer packages [39,40,41]. The *p* ≤ 0.05 was defined statistically significant.

## 3. Results

### 3.1. Demographics

**Figure 1:**
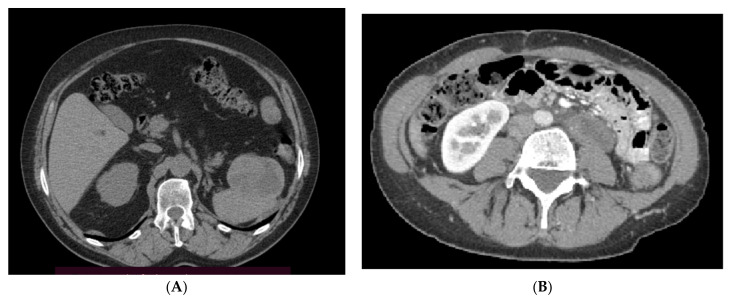
Patient with metastatic retroperitoneal PEComa of the kidney (**A**) and PEComa NOS (**B**) with large pelvic and intraperitoneal tumors.

**Figure 2:**
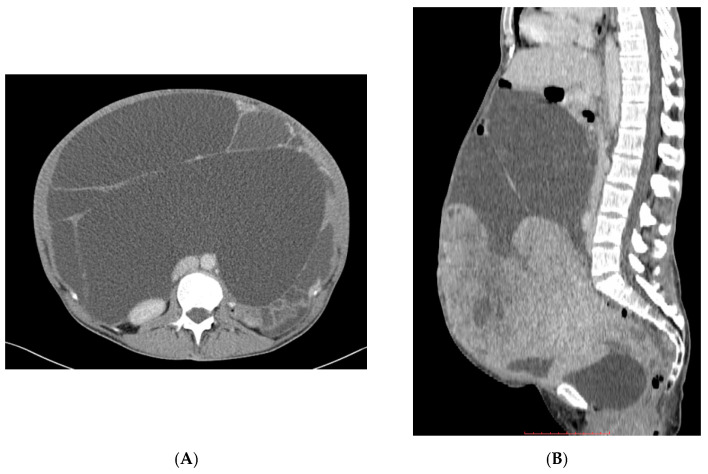

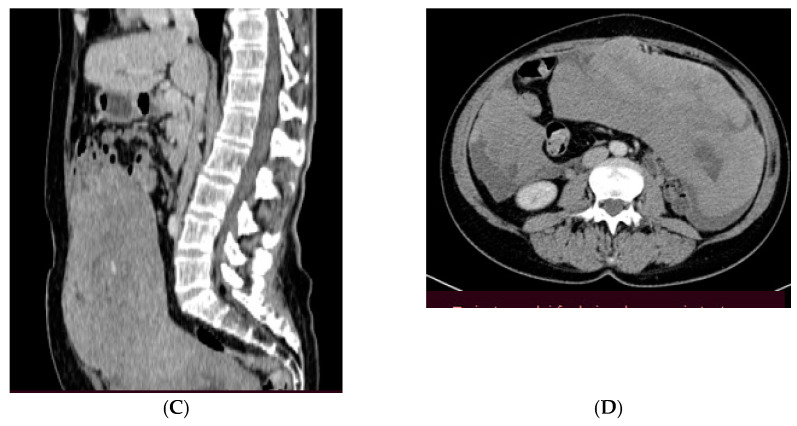
Patient with massive peritoneal effusion due to retroperitoneal LAM (**A**,**B**) and response to treatment with sirolimus (**C**,**D**).

Fifteen patients with metastatic PEComa started sirolimus treatment between March 2011 and August 2020; 7 had unresectable and 8 metastatic disease at the start of treatment (Figure 1). Forty-seven percent of patients had prior surgery outside of our hospital for the primary tumor, while 1 underwent radical surgery in MSCNRIO and relapsed later. 8/8 patients developed metastases in the abdomen and 5 in the lungs. Eleven patients were treated with sirolimus up-front (Figure 2), while 4 received doxorubicin-based chemotherapy and sirolimus as further line therapy. The median age of the patients was 50 (IQR: 34–60) years. The majority were women (80%). Primary sites of origin were gynecological or abdomen/retroperitoneal space. Liver, lung, and retroperitoneal space were the most frequent metastatic sites (Figure 1 and Figure 2; Table 1). None of our patients was diagnosed with tuberous sclerosis complex or presented any signs or symptoms of this disease.

### 3.2. Treatment

**Figure 3:**
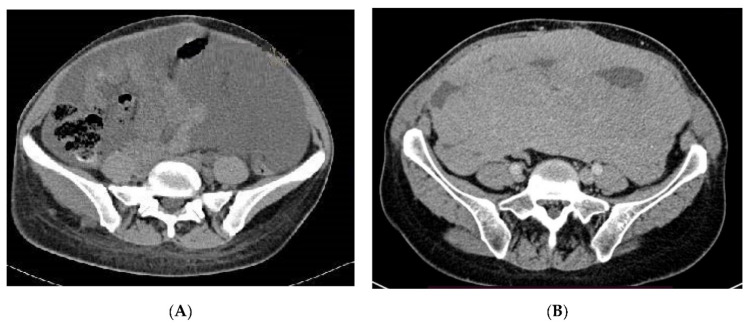
Patient with peritoneal effusion due to retroperitoneal LAM (**A**) and fluid resorption on sirolimus treatment (**B**).

Sirolimus was started at 2–6 mg per day (qd) orally once daily and continued according to patient tolerability as per attending physician choice (Table 1). During therapy, 10 patients required dose reductions, 11 patients received sirolimus in the first-line metastatic setting, and 4 patients were previously treated with chemotherapy. The median dose recommended was 3 mg daily and the median duration of treatment was 25 months (range 5–63 months), mean 29 months. All patients received clinical benefits and symptoms palliation. In most patients, stabilization of the disease (SD) was achieved, and 73% of patients achieved an objective response (ORR) (Figure 2 and Figure 3).

Treatment toxicity was managed by dose reductions or/and treatment interruption. Ten patients had dose reductions and 2 had dose interruptions. Treatment toxicities were generally mild, manageable, and responded well to dose reductions. In 6 patients, prolonged hypercholesterolemia and hypertriglyceridemia resulted in dose reductions along with atorvastatin and fenofibrate treatment. The highest total cholesterol level recorded was 358 mg/dL. Two other cases with erosive mouth mucositis, one with upper extremity edema and one with reduced glomerular filtration rate on sirolimus treatment, required dose reductions. One of the patients with mucositis also presented prolonged G1 hyperbilirubinemia that resolved after dose reduction. Patients with diarrhea accompanied by abdominal pain required dose interruptions. No hypersensitivity reactions, including anaphylactic or anaphylactoid reactions, angioedema, exfoliative dermatitis, and hypersensitivity vasculitis or lymphedema, were reported. Pulmonary embolism, pulmonary hemorrhage, pancreatitis, nephrotic syndrome were also not reported. No treatment-related deaths were recorded.

### 3.3. Efficacy

**Figure 4:**
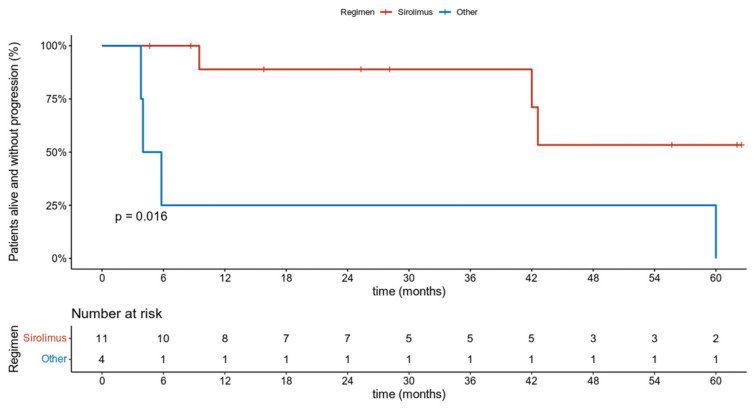
Progression-free survival in PEComa patients receiving sirolimus (**red line**) or chemotherapy (**blue line**).

Median progression-free survival (PFS) in patients receiving sirolimus as first-line therapy was not reached (95% CI: 42.0-NA) and were 42.6 months (95% CI: 21.9-NA) when considering this treatment regardless of the line. At the same time, the median PFS for the chemotherapy in the first line was 4.9 months (95% CI: 3.8–NA) (Figure 4). There was a single case of objective response (OR) during chemotherapy. At the time of analysis, sirolimus treatment was discontinued in 12 patients and 3 patients died. The primary reason for discontinuation was PD in 6 patients and 3 patients died due to disease progression after 29 and 32 months since metastatic disease diagnosis. After a median follow-up of 55.7 (95% CI: 32-NA) months since the start of first-line therapy, the 5 yr OS was 83% (CI 95% 58–100) for first-line sirolimus patients and 65% (95% CI: 39–100) for the whole group. Three patients were remaining on treatment at the time of data cut-off. Following PD 2 patients received further systemic treatment.

## 4. Discussion

Before molecular discoveries, treatment of metastatic PEComa was ineffective as there was no proven role for chemotherapy, and the prognosis for patients with metastatic disease was poor [4,6,42]. Pharmacological inhibition of mTOR signaling is expected to result in significant clinical activity in PEComa patients and radiological responses to sirolimus are to be observed in most patients [43]. In a group of patients with such limited therapeutic options, the development of targeted therapy is critical [4,6,42]. To date, this is the largest reported series of patients with PEComa treated with sirolimus in real-world practice. Inhibition of hyperactivated mTORC1, which results from loss of the *TSC1*/*TSC2* tumor suppressor complex, is a specific molecular target for PEComas therapy. We have observed significant clinical responses in patients treated with sirolimus, including the longest ongoing response of greater than 62 months of duration. Identification of specific other molecular/genetic alterations in this sarcoma subtype could lead to the development of more effective therapies for this challenging group of diseases in the future. 

Until the introduction of mTOR inhibitors, the only option for the treatment of metastatic PEComa was chemotherapy, mostly doxorubicin-based regimens. No formal recommendations or guidelines were provided for first-line chemotherapy in PEComa, and treatment strategies differ between cases and hospitals [14]. The first evidence of mTORC1 activation in PEComa was delivered by the study of 15 cases in which the absence of AKT phosphorylation was shown [14]. Subsequently, high levels of phospho-p70S6K were reported in PEComa cells [18]. It was concluded that the presence of high levels of S6 phosphorylation is to correlate with a high likelihood of disease control with an mTOR inhibitor [44]. In a systematic review of AML and sirolimus, in total, 94 patients were included. The review covered four prospective nonrandomized studies. In general, the results reported for AML only population were consistent with our general PEComa study. In the review, ORR in AML was 46.8% in the first year, 43.5% in the second year [45]. In comparison to the review, we reported significantly longer follow-up of the patients. In concordance to the reported cohort of AML, where the volume of the tumors decreased 53% after 12 months of therapy, we reposted significant responses (Figure 1) [20]. A new derivative of sirolimus nab-sirolimus (albumin-bound) was recently tested in the single-arm AMPECT study of 31 malignant PEComa patients—nab-sirolimus, an injectable a nanoparticle albumin-bound (*nab*^®^) sirolimus with a mean particle size of approximately 100 nm. 100 mg/m^2^ of the drug was administered intravenously weekly for 2 weeks, followed by a week of rest until PD or unacceptable toxicity. Median PFS was 8.9 months, OS-40.8 months, while ORR-39% (95% CI: 22–58). Responses were durable, with 50% of patients having an ongoing response for more than 25 months. Nevertheless, nab-sirolimus is still not available outside of clinical trials [46,47,48]. A tumor-agnostic registrational trial in cancers with *TSC1* or *TSC2* inactivating alterations is expected. FDA approval for nab-sirolimus in advanced PEComa is expected on 26 November 2021.

As phase 1 study with sirolimus in patients with solid tumors defined a maximum tolerated dose of 6 mg and pharmacokinetic analysis showed that drug exposure increased proportionally with dose [49] we have used median dosing of 3 mg/day. It was also suggested that in patients who do not initially respond to dose escalation would represent a reasonable treatment approach, best of all in conjunction with drug level monitoring [30], but such approach is hard to achieve in routine practice outside clinical trials. Safety of the treatment in routine practice observed by us was typical, without novel serious adverse events. The most common known sirolimus-related AEs reported are stomatitis, respiratory infections, and hyperlipidemia [45]. It was proven that dose-adjusted sirolimus may be used with a prolonged clinical benefit. It was suggested that in cases with high toxicity, pharmacokinetic sirolimus measurement should be used as this drug has a narrow therapeutic window. It should also be remembered that sirolimus blood levels may be influenced by CYP3A4 polymorphisms and subsequently cytochrome-based drug interactions [37]. CYP3A4 inhibition with ciprofloxacin and grapefruit juice has also been reported to increase sirolimus levels [49].

PEComa patients’ treatment should be managed in reference sarcoma centers with a medical oncology team and, after, a multidisciplinary tumor board. The first step towards best treatment selection is a pathology report. Referral centers in sarcoma pathology are indispensable for a high level of histological diagnosis [50,51]. Since PEComas are almost always immunoreactive for smooth muscle (actin, desmin, caldesmon) markers, as well as melanocytic (HMB-45, melan-A, MiTF) markers, this characteristic immunohistochemical profile provides accurate diagnosis [4,6,42,52]. PEComa should be considered as malignant when it reaches size ≥5 cm, and has concomitant characteristics of mitoses ≥1/50 HPF, significant nuclear atypia, necrosis, and lymphovascular invasion [53]. Tumors larger than 5 cm in diameter with micro-hemorrhages, necrosis, and capsular invasions should also be considered to have malignant potential [54]. At the same time, imaging features of PEComas are nonspecific and may mimic other benign and malignant tumors, so an experienced radiologist is also required [55]. Primary PEComa tumors are usually well-circumscribed, heteroechoic on ultrasound examination, hypodense to isodense on CT with intense contrast enhancement, hypointense to isointense in comparison to skeletal muscle on T1-weighted imaging, and heterogeneously hyperintense on T2-weighted imaging with significant gadolinium enhancement on MRI [56]. Given the tumor characteristics and response pattern of this PEComa, there could be a role for incorporation of Choi criteria into CT evaluation on treatment as in the case of other sarcoma subtypes, including gastrointestinal stromal tumor (GIST) [30,57].

Based on the increasing amount of data reported by us and others, sirolimus (or other mTOR inhibitors) is expected to be the best first-line therapy in advanced and metastatic PEComa. PEComa patients, if diagnosed in community-based hospitals, should be referred to sarcoma centers for mTOR inhibitor therapy or clinical trial enrolment. At the same time, due to the rarity and different sites of presentation, the best multidisciplinary management of PEComa is still a matter of debate, also in terms of the timing of surgery and the need for neoadjuvant or adjuvant treatment [58]. The question of which therapeutic options to consider in PEComa following disease progression is also open, as the mechanism of mTOR inhibitors resistance is until now poorly characterized. More extensive molecular research on PEComa drug resistance is needed.

## Figures and Tables

**Table 1:** Summary or patients characteristics.

Patient No	Primary Site	PEComa Subtype	Previous Surgery	Line of Treatment	Previous Chemotherapy	Duration of Sirolimus Response	Best Response (RECIST)	Sirolimus Dose
1	Retroperitoneal	AML	Yes	1	NA	55.7	PR	4 mg qd
2	Retroperitoneal	NOS	No	2	ADIC	32	PR	3 mg qd
3	Abdomen	NOS	Yes	1	NA	9.5	SD	6 mg qd
4	Genital	NOS	Yes	1	NA	62.1	CR	4 mg qd
5	Genital	NOS	No	2	ADIC	16	PR	5 mg qd
6	Genital	NOS	Yes	2	DDP + DOX	9.2	SD	4 mg qd
7	Retroperitoneal	NOS	No	1	NA	42	PR	4 mg qd
8	Visceral	AML	Yes	1	NA	42.6	CR	4 mg qd
9	Trunk	LAM	No	1	NA	25.3	SD	4 mg qd
10	Retroperitoneal	LAM	No	1	NA	28.1	PR	4 mg qd
11	Abdomen	NOS	Yes	4	EP, ADIC, Gemcitabine	21.9	SD	6 mg qd
12	Trunk	LAM	Yes	1	NA	62.5	CR	4 mg qd
13	Trunk	NOS	Yes	1	NA	15.8	PR	3 mg qd
14	Trunk	LAM	No	1	NA	8.7	PR	3 mg qd
15	Retroperitoneal	NOS	No	1	NA	4.7	PR	2 mg qd

## Data Availability

All data generated or analyzed during this study are available upon reasonable request upon DTA consent.
